# Metabolite Formation by Fungal Pathogens of Potatoes (*Solanum tuberosum* L.) in the Presence of Bioprotective Agents

**DOI:** 10.3390/ijerph20065221

**Published:** 2023-03-22

**Authors:** Aleksandra Steglińska, Michael Sulyok, Regina Janas, Mieczysław Grzesik, Wiktoria Liszkowska, Dorota Kręgiel, Beata Gutarowska

**Affiliations:** 1 Department of Environmental Biotechnology, Lodz University of Technology, Wólczańska 171/173, 90-950 Łódź, Poland; wiktoria.liszkowska@dokt.p.lodz.pl (W.L.); dorota.kregiel@p.lodz.pl (D.K.); beata.gutarowska@p.lodz.pl (B.G.); 2 Interdisciplinary Doctoral School, Lodz University of Technology, Żeromskiego 116, 90-924 Łódź, Poland; 3 Department of Agrobiotechnology (IFA-Tulln), Institute of Bioanalytics and Agro-Metabolomics, University of Natural Resources and Life Sciences, Vienna, Konrad-Lorenz-Strasse 20, 3430 Tulln, Austria; michael.sulyok@boku.ac.at; 4 The National Institute of Horticultural Research, Konstytucji 3 Maja 1/3, 96-100 Skierniewice, Poland; regina.janas@inhort.pl (R.J.); mieczyslaw.grzesik@inhort.pl (M.G.)

**Keywords:** biocontrol, lactic acid bacteria, garlic extract, *Metschnikowia* yeasts, potato physiology, mycotoxins, phytotoxins

## Abstract

The potato is a crop of global importance for the food industry. This is why effective protection against pathogens is so important. Fungi as potato pathogens are responsible for plant diseases and a significant reduction in yields, as well as for the formation of mycotoxins. This study focuses on the effect of three natural biocides, yeast *Metschnikowia pulcherrima*, lactic acid bacteria *Lactiplantibacillus plantarum*, and aqueous garlic extract, on the improvement of the physiology of planted potato tubers and the reduction in mycotoxin formation. The secondary metabolites produced by the fungal pathogens of genera *Fusarium, Alternaria, Colletotrichum, Rhizoctonia*, and *Phoma* in the presence of these biocontrol agents were compared to profiles obtained from contaminated potatoes. Analysis of liquid chromatography coupled with tandem mass spectrometry data showed the presence of 68 secondary metabolites, including the mycotoxins: alternariol, alternariol methyl ether, altertoxin-I, aurofusarin, beauvericin, diacetoxyscirpenol, enniatin B, and sterigmatocystin. The studies showed that the applied biocontrol agents had a positive effect on the physiological parameters of potatoes (including root growth, stem growth, gas exchange, and chlorophyll content index) and on the reduction in the production of mycotoxins and other secondary metabolites by *Fusarium*, *Alternaria*, and *Phoma*.

## 1. Introduction

Potato (*Solanum tuberosum* L.) tubers are the world’s fourth most-consumed crop, cultivated in 160 countries, with 322 Mt of yield annually [1]. Poland, Germany, France, Romania, the Netherlands, and Belgium are the leading countries in potato production in the European Union with 55.3 Mt of tubers produced in total [2]. Crop pathogenic microorganisms are responsible for diseases, which cause reductions in yield and the quality of potatoes (worldwide loss estimate is 17.2%). This presents a great economic risk and a threat to global food security [3]. The most common fungal pathogens of potato tubers include the *Alternaria* spp., the *Fusarium* spp., *Phoma exigua*, *Helminthosporium solani*, *Colletotrichum coccodes*, *Rhizoctonia solani*, and *Phytophthora infestans* [4]. Some of them are able to produce mycotoxins [5]. 

Mycotoxins are low-molecular-weight toxic secondary metabolites (the majority are <700 Da) produced by certain fungi (*Fusarium*, *Alternaria*, *Penicillium*, *Aspergillus*, and other species), naturally present in foodstuffs of plant origin such as seeds, fodder, cereal, fruit, and hazelnuts. More than 400 mycotoxins have already been identified, some of which can be produced by more than one fungus genus. Certain species of fungi are able to synthesise several mycotoxins [6]. The most common mycotoxins include aflatoxins (AFB_1_, AFB_2_, AFG_1_, AFG_2_, and AM_1_), ochratoxin A (OTA), zearalenone (ZEN), deoxynivalenol (DON), T-2 and HT-2 toxins, patulin (PAT), and fumonisins (FB1 and FB2) [7]. Mycotoxins produced by fungi can be detrimental to both humans and animals with carcinogenic, neurotoxic, hepatotoxic, and teratogenic effects [8,9]. In European Union countries, Commission Regulation (EC) No. 1881/2006 of 19 December 2006 determines the maximum permissible mycotoxin level in certain commodity groups [10]. There are only few studies which have described mycotoxin occurrence in potato plants, mainly concerning the presence of mycotoxins produced by the *Fusarium* spp. [11,12,13]. There is a lack of research on the production of mycotoxins on potatoes by genera of other fungi. Most available research focuses on assessing mycotoxin contamination in maize, wheat, soybean, rice, and barley [7,14]. 

Most mycotoxins are heat-stable and difficult to remove from contaminated products. The threat to human and animal health is associated with their high ability to bioaccumulate in crop plants [8]. Mycotoxins occur mainly in agricultural fields, from sowing to harvesting; however, they can also be produced during inappropriate storage conditions [15]. Potato storage conditions, such as relative humidity (80–90%), temperature, long storage time (up to 8 months), and soil contamination are favourable to mould growth [16]. 

An effective way to reduce mycotoxin contamination in seed potatoes at an early stage is to prevent the development of mycotoxin-producing fungi. This can be achieved through various biological approaches, e.g., the use of microorganisms and their metabolites, plant extracts, or combined methods with certain synthetic compounds [8]. In our previous research, we described the inhibition of phytopathogen growth using three biological control agents: lactic acid bacteria [17], yeast *Metschnikowia pulcherrima* [18], and garlic water extract [19]. 

The potential application of lactic acid bacteria (LAB) as a biological control agent against plant pathogens has been previously described for several pathogens ranging from the *Fusarium*, *Alternaria*, *Colletotrichum*, and *Rhizoctonia* to the *Phoma* species [17,20,21,22]. Lactic and acetic acid, hydrogen peroxidase, cyclic dipeptides, phenyllactic acid, and 3-hydroxy fatty acids are mainly responsible for the antimicrobial activities of LAB [23,24,25]. In addition to direct antifungal activity, some LAB strains have demonstrated the ability to convert already produced mycotoxins into less or nontoxic compounds [4,6] or to remove them entirely by binding to LAB cell walls, both living and dead. The mechanism of mycotoxin removal is based on the adsorption abilities linked to the presence of peptidoglycans, proteins, and polysaccharides in LAB cell walls [6,26]. 

Yeast *Metschnikowia pulcherrima* is another biological control agent that we used for potato protection against mycotoxins produced by fungi. These yeasts are the natural microbiota of fruit, fruit juices, flowers (nectar), and flower insects [27,28]. Their main antimicrobial activity is connected to the secretion of pulcherriminic acid that forms pulcherrimin—a red complex with iron ions. This nonenzymatic reaction removes iron, an essential element for pathogen growth [29]. The published data provide information about the antifungal activity of yeast *M. pulcherrima* against potato pathogens from the *Fusarium*, *Alternaria*, *Rhizoctonia*, *Phoma*, and *Colletotrichum* species [18,30,31]. The composition of the culture medium and the conditions for the cultivation of *M. pulcherrima* yeasts and selected lactic acid bacteria used as biopreservatives were developed in earlier studies conducted by Steglińska et al. [17,18,32,33]. 

In this work, we evaluated the effect of aqueous garlic extract as a plant biopesticide against mycotoxin-producing fungi that infect potatoes. Several works confirmed the antifungal activity of garlic extracts on *Alternaria*, *Rhizoctonia*, *Fusarium*, *Colletotrichum*, and *Phoma* fungi [19,34,35,36]. The antimicrobial activity of garlic extracts is linked to the production of organosulphur compounds such as allicin, ajoenes, diallyl disulphide, methyl allyl disulphide, and others [37].

Due to the global production of potatoes, the climatic change favouring the development of potato phytopathogens, the high degree of infestation during storage, and the withdrawal of many pesticides from the market due to their accumulative and toxic abilities, new ecological methods of protection need to be developed. Therefore, selected natural active ingredients of biopreparations based on microorganisms and plants may be a solution. Their high effectiveness in inhibiting mould growth was proven in earlier studies [17,18,19]. There are no data, however, considering the effect of biopreparations on the formation of mycotoxins by moulds on potato tubers, as well as on the growth and physiological activity of potatoes. Therefore, the aim of this research was to evaluate the profiles of secondary metabolites in potato seeds in response to different fungal phytopathogens. In addition, the alteration of these profiles in the presence of selected biocontrol agents of microbial and plant origin was evaluated. Hence, we assessed the possibility of using *M. pulcherrima* yeast, lactic acid bacteria, and aqueous garlic extract as preservatives for potatoes. Additionally, the impact of the use of these biopreparations on the growth and physiological activities of potatoes was assessed. 

## 2. Materials and Methods

### 2.1. Biological Material Used in the Research

#### 2.1.1. Plant Material

The experiments were performed on the Impresja variant of potato tubers (*Solanum tuberosum* L.). This is an early potato, widespread in Poland with potential for cultivation in different countries. Potatoes were obtained from the Hodowla Ziemniaka Zamarte Sp. z o. o. (IHAR Group, Kamieńsk Krajeński, Poland; 53°35′58″ N, 17°28′54″ E). 

#### 2.1.2. Potato Phytopathogens

Five fungal potato phytopathogens, *Fusarium sambucinum* DSM 62397, *Alternaria tenuissima* DSM 63360, *Colletotrichum coccodes* DSM 62126, *Phoma exigua* DSM 62040, and *Rhizoctonia solani* DSM 22843, were used in the experiments. The strains were purchased from the German Collection of Microorganisms and Cell Cultures GmbH (DSMZ, Braunschweig, Germany). All strains were activated on Potato Dextrose Agar (PDA; Merck, Darmstadt, Germany) and stored at 4 °C. Pathogen suspensions were prepared from the pure cultures on the PDA agar plates and adjusted to a final concentration of 10^6^ CFU/mL in saline solution (0.85% NaCl). 

For fungal metabolite analysis (Section 2.3), samples of fungal cultures on Malt Extract Agar (MEA; Merck, Darmstadt, Germany) plates and potatoes infected with phytopathogens were prepared.

#### 2.1.3. Lactic Acid Bacteria Biopreparation (LpB)

The bacterial strain *Lactiplantibacillus plantarum* KB2 LAB 03 isolated from sauerkraut was used to obtain bacterial preparations against potato phytopathogens with a previously described spectrum of activity [17]. Lactic acid bacteria were identified using molecular methods based on sequencing of the 16S rRNA gene fragment. Genomic DNA was isolated using the Genomic Mini kit (A&A Biotechnology, Poland), according to the methodology provided by the manufacturer. The reaction mixture was prepared in a volume of 50 µL containing 25 µL of REDTaq™ ReadyMix™ (Sigma, Saint Louis, MO, USA) polymerase (1.5 units), 0.4 µL of each primer solution (at a concentration of 100 µM), 25 µL of water, and 20 ng of template DNA. The 16S rRNA gene was amplified by PCR in the MJ Mini Gradient Thermal Cycler (Bio-Rad, Hercules, CA, USA) using the universal primers 5′–AGAGTTTGATCCTGGCTCAGGA–3′ (forward) and 5′–GGAGGTGATCCAGCGGC–3′ (reverse). The cycle consisted of pre-denaturation at 94 °C for 2 min, denaturation at 94 °C for 1 min, primer annealing at 50 °C for 1 min (34 repetitions), elongation at 72 °C for 3 min, and final elongation at 72 °C for 3 min. The obtained PCR products were analysed by 1% (*w/v*) agarose gel electrophoresis. The amplified PCR products were purified using the Clean-Up AX kit (A&A Biotechnology, Gdansk, Poland) and then subjected to a sequencing reaction at an external company—GENOMED S.A. (Warsaw).

The strain was activated and then cultured for further experiments on an acid–whey-based medium supplemented with 0.8% yeast extract (BTL, Łódź, Poland), 1.4% peptone K, 0.2% ammonium citrate (Chempur, Poland), 0.2% dipotassium phosphate (Chempur, Poland), 0.5% sodium acetate (Chempur, Poland), 0.02% magnesium sulphate heptahydrate (Chempur, Poland), and 0.005% magnesium sulphate tetrahydrate (Chempur, Poland) for 48 h at 30 °C [17]. The acid–whey was obtained from JOGO–Łódź Dairy Cooperative (Kraszewo, Poland). 

#### 2.1.4. Metschnikowia Pulcherrima Yeast Biopreparation (MpB)

The strain *Metschnikowia pulcherrima* TK1 isolated from strawberry flowers was used to obtain yeast preparations against potato phytopathogens with a previously described spectrum of activity [18]. A pure culture of yeast was identified by MALDI-TOF MS analysis, described in detail in Steglińska et al. [18]. Briefly, a 24 h culture of yeast cultured on an MEA plate (Merck, Darmstadt, Germany) was analysed with the AXIMA-iD Plus Confidence MALDI-TOF MS system (Kratos Analytical Ltd. and Shimadzu Corporation, Kyoto, Japan) and SARAMIS PREMIUM software (Spectral Archive And Microbial Identification System, bioMérieux, Marcy l’Étoile, France). The direct formic acid smear method was used. The yeast colony was spread on an analytical plate with a sterile loop. After that, 0.5 µL of 25% formic acid was spotted onto cells, mixed, and left until almost dry. Then, 1 µL of saturated α-cyano-4-hydroxycinnamic acid solution in an acetonitrile, ethanol, and water (1:1:1; *v/v*) mixture containing 3% trifluoroacetic acid was added onto the yeast cells, mixed, and dried. The mass spectra were obtained and processed using Launchpad 2.9 software (Kratos Analytical Ltd. and Shimadzu Corporation, Kyoto, Japan) in the SARAMIS linear positive mode, with a laser frequency of 50 Hz in a mass-to-charge ratio (*m/z*) range from 2000 to 20,000 Da (laser power: 90; 200 per sample; five shots accumulated per profile) for each mass spectrum. *E. coli* DH5 (TAKARA BIO INC.) cells were used as a calibrator of the AXIMA-iD Plus Confidence MALDI-TOF MS system and internal control of the identification process, in accordance with the recommendations of the manufacturer. 

The strain was activated and then cultured for further experiments on an acid–whey-based medium supplemented with 0.13% yeast extract, 0.25% peptone, and 1% glucose for 72 h at 25 °C on a shaker (Unimax 1010, Heidolph, Germany) at 160 rpm [18]. The acid–whey was obtained from JOGO–Łódź Dairy Cooperative (Kraszewo, Poland). 

#### 2.1.5. Aqueous Garlic Extract Biopreparation (GB)

Garlic (*Allium sativum* L.) was obtained from Dary Natury Sp. z o. o., Grodzisk, Poland. The aqueous plant extract was prepared by pouring 50 g of finely ground material in 500 mL of water at 100 °C, which was left covered without stirring for 1 h. After that, the garlic extract was sonicated (40 kHz, 25 °C, 30 min) and filtered under reduced pressure [19]. 

### 2.2. Sample Inoculation with Phytopathogens and Application of Bioprotective Agents

“Impresja” seed potatoes were rinsed in sterile distilled water and left to dry in air. Then, 5 mm wide and 5 mm diameter cuts were made into each potato with a cork bore. Potato biopreparations were made according to Section 2.1.3, Section 2.1.4 and Section 2.1.5. Samples were immersed in LpB, MpB, and GB for 5 min and then left to dry. After that, 20 µL of each fungal suspension was applied to the cuts. Control potatoes were only inoculated with pathogen suspensions. Samples were incubated for 14 days at a temperature of 25 °C.

### 2.3. Fungal Metabolite Analysis by LC–MS/MS 

For fungal metabolite analysis, plugs were cut from the investigated potatoes and MEA plates with a sterile cork bore, and then analysed according to the validated method described in detail by Sulyok et al. [38] for LC–MS/MS analysis of >500 mycotoxins and other secondary metabolites in food crops. Samples weighing 1–3 g were extracted with 5 or 10 mL (depending on the weight of the sample) by extraction solvent (acetonitrile/water/formic acid 79:20:1, *v/v/v*) for 90 min on a GFL rotary shaker (GFL, Burgwedel, Germany). After extraction, aliquots of the prepared extracts were diluted 1:1 using a dilution solvent (acetonitrile/water/formic acid 20:79:1, *v/v/v*). Then, 5 µL of the diluted extracts were injected into the column of the liquid chromatography apparatus, Agilent 1290 Series HPLC System (Agilent Technologies, Waldbronn, Germany), coupled with a QTrap 5500 m (Sciex, Foster City, CA, USA) equipped with a Turbo Ion Spray ESI source. The compounds were separated using a Gemini® C18 column, 150 × 4.6 mm i.d., 5 μm particle size from Phenomenex (Torrance, CA, USA) protected by a C18 security guard cartridge, 4 × 3 mm i.d. (Phenomenex, Torrance, CA, USA) at 25 °C. The eluent was a methanol/water gradient containing 1% acetic acid and 5 mM ammonium acetate. The elution rate was 1 mL/min. Scheduled multiple reaction monitoring (sMRM) in both negative and positive mode after two separate chromatographic runs per sample was performed for data acquisition. Confirmation of the identity of the metabolites was obtained through the comparison of retention time and intensity ratio to a multianalyte standard (SANTE/12089/2016). External quantification was performed on the basis of the linear calibration curves from serial dilutions of a multianalyte standard. The accuracy of the method is verified on a routine basis by participation in a proficiency testing scheme organised by BIPEA (Genneviliers, France). Currently, the rate of satisfactory results of −2 < z < 2 is 95.6% with >1900 results submitted. Limits of detection (LOD) and limits of quantification (LOQ) are presented in Table S2.

### 2.4. Growth and Physiological Parameter Analysis

#### 2.4.1. Methods of Plant Cultivation

For greenhouse experiments, a mix of phytopathogen suspensions was used to inoculate potatoes after biopreparation treatment. After incubation, potatoes were subjected to negative selection and cultivated in a ventilated greenhouse (20 °C), in modified plant microcosms with dimensions 30 × 40 × 10 cm (height × width × depth, respectively). The front walls of plant microcosms were made from transparent glass, which enabled constant observation of tuber germination, root growth and quality, and the dynamics of stem growth [39]. The microcosms were filled with 10 L of standard horticultural substrate mixed with a universal complete fertiliser containing macro- and microelements (YaraMila Complex; Yara) at a dose of 2 kg/m^3^. The plants were watered with tap water as needed throughout the growing season [40,41]. 

#### 2.4.2. Assessment of Growth and Physiological Activity Parameters

After potatoes were planted in microcosms, the growth and physiological activity of the plants were checked every 2 days for the first 24 days, and every 6 days for the remaining period [41]. The following parameters were measured:

- Biological condition of the seed potatoes—their vigour, turgor, rotting, and infection by phytopathogens [42];
- The percentage of germinating tubers [42];
- Growth kinetics of the plant, by measuring the length of shoots every 2 days during the first 24 days, and every 6 days for the remaining period [43];
- Quality of the shoots on a five-point valuation scale, where 5 indicates shoots growing well, full of vigour, while 1 indicates dried out shoots [44];
- Quality and growth kinetics of root, by measuring root length every 2 days until they reached 30 cm [42];
- Growth kinetics of the root system, by assessing every 2 days the percentage of the soil profile area filled by the roots [45];
- Quality of plants on a five-point rating scale, where 5 indicates plants with high vigour, coloured and growing properly, and 1 indicates dried out plants [8];
- Intensity of gas exchange (transpiration, net photosynthesis, intercellular CO_2_ content, and stomata conductivity), measured with a TPS-2 apparatus (PP Systems, USA) in the highest positioned fully developed leaves, during the period of most dynamic plant growth [39];
- Index of chlorophyll content, measured with a Minolta SPAD-502 apparatus (Japan) in the highest positioned fully developed leaves, during the period of most dynamic plant growth [44].

In each experimental variant, the plants were grown in three repetitions of four plants in each. This means that 12 plants were evaluated in each variant.

### 2.5. Statistical Analysis

Statistical analysis was performed with Statistica 13.1 (Statsoft, Tulsa, OK, USA). All measurements of plant growth and physiological activity were performed in triplicates of four plants in each repetition, and the results are reported as the mean ± standard deviation. For each value, a one-way ANOVA with Duncan’s multiple ranges was performed to determine significance at a confidence level of *p* < 0.05. Measurements of secondary metabolites were compared using a one-way analysis of variance (ANOVA) at a significance level of 0.05. When a statistical difference was detected (*p* < 0.05), the means were compared using Tukey’s post hoc procedure at a significance level of 0.05.

## 3. Results and Discussion

### 3.1. Fungal Metabolite Analysis by LC–MS/MS

In our work, the secondary metabolite profile of *Fusarium sambucinum*, *Alternaria tenuissima*, *Colletotrichum coccodes*, *Phoma exigua*, and *Rhizoctonia solani* was determined by LC–MS/MS. Fungi were cultivated on Malt Extract Agar (MEA) plates as pure cultures on the potatoes. The effect of the use of biological treatments on the profile of metabolites (especially mycotoxins), including aqueous garlic extract biopreparation (GB), *Metschnikowia pulcherrima* TK1 biopreparation (MpB), and *Lactiplantibacillus plantarum* KB2 LAB 03 (LpB) biopreparation was assessed. The results are presented in Table 1, Table 2, Table 3, Table 4 and Table 5 and Table S1. In total, 68 different metabolites were quantified by LC–MS/MS. Among them were 18 *Fusarium* metabolites (fusaric acid (FSA), acuminatum B and C, aurofusarin (AUR), beauvericin (BEA), bikaverin, chrysogin, cyclosporin A, B, D and H, deoxygerfelin, diacetoxyscirpenol (DAS), enniatin B (Enn B), equisetin, gibepyron D, sambutoxin and siccanol; eight from the *Alternaria* species: 4-hydroxyalternariol, alteichin (ALTCH), alternariol (AOH), altersetin (ALN), alternariolmethylether (AME), altertoxin-I (ATX 1), radicinin (RAD), and tentoxin (TX)), 14 derived from other fungi (asterric acid, beauvericin A, cytochalasin B and E, isosulochrin, monocerin, rosellichalasin, secalonic acid D, sterigmatocystin, sulochrin, terragine, trichodimerol, trichotetronine, and xanthoquinoidin A1), three bacterial metabolites (dinactin, monactin, and nonactin), eight of plant origin (abscisic acid, daidzein, daidzin, genistein, genistin, glycitein, glycitin, and solanine), two from lichens (vulpinic acid and lecanoric acid), and 15 unspecific ones (brevianamide F, citreorosein, cordycepin, cyclo(L-Leu-L-Pro), cyclo(L-Pro-L-Tyr), cyclo(L-Pro-L-Val), emodin, endocrocin, fallacinol, fellutanine A, iso-rhodoptilometrin, rugulusovin, tryptophol, and violaceol I and II) (Table 1, Table 2, Table 3, Table 4 and Table 5; Table S1). 

The results for secondary metabolites of *Fusarium sambucinum* growing on MEA plates and potatoes are presented in Table 1. Eight metabolites were determined in a pure *F. sambucinum* culture on MEA plates of which beauvericin, chrysogin, and diacetoxyscirpenol are recognised as *Fusarium* mycotoxins [15,46,47] and two more components (acuminatum B and C) are recognised as specific for the *Fusarium* species. Paciolla et al. [48] reported phytotoxic activity (by disruption of the ascorbate system) of bauvericin in tomato protoplasts. This compound has also exhibited cytotoxic properties on mammalian cells by affecting the cell membranes and mitochondria [49]. Diacetoxyscirpenol belongs to the trichothecene group of mycotoxins. Their toxic activity results from blocking protein synthesis [15]. For potatoes infected with *F. sambucinum*, the metabolite profile was different from the pure culture on MEA. It consisted of 18 compounds, including four mycotoxins/phytotoxins (aurofusarin, beauvericin, sambutoxin, and fusaric acid) and two more specific compounds (bikaverin and deoxygerfelin) (Table 1). Sambutoxin has been already determined in rotten potato tubers infected with *F. sambucinum* [50]. Kim and Lee work confirmed its toxic activity in a rat-feeding test [51]. Fusaric acid was reported as a phytotoxin that contributed to the pathogenesis of *Fusarium* in tomatoes by reducing the activity of antioxidant enzymes and increasing reactive oxygen species (ROS). It eventually led to plant cell death [52]. We observed a possible interesting interaction between GB and *Fusarium*, which resulted in a several-fold increase in the amount of produced fusaric acid. The LC–MS/MS analysis did not show the presence of other mycotoxins specific to *Fusarium* spp. commonly found in the published data [12,13,46], such as DON, nivalenol (NIV), or ZEN in any of the potato samples inoculated with *F. sambucinum*. Furthermore, rubrofusarin, usually occurring with aurofusarin [53], was not marked in our research. The fact that twofold more components were identified in potato samples than in MEA may be the result of the presence of natural potato microflora, differences in fungi metabolism depending on the available substrates for growth, and some possible cross-contamination in the potato samples which might originates from the soil. Similarly, Kokkonen et al. [54] observed the distinctive influence of the culture medium on mycotoxin production by some *Penicillium* species. Westphal et al. [55] described significant differences in the metabolite profile of four *Fusarium* species, depending on the manufacturer of the Potato Dextrose Agar (PDA), a commonly used medium for fungi cultivation. 

Overall, the incorporation of the biological treatment had a positive effect on reducing the fungal contamination of potatoes with selected *Fusarium* mycotoxins (Table 1). Compared to the biologically untreated potatoes, the presence of aurofusarin was not detected in potato samples treated with GB and MpB. Aurofusarin is a golden-yellow pigment with described genotoxic, cytotoxic, and oxidative stress-inducing abilities in human colon cells [56]. Fusaric acid and sambutoxin were absent in potatoes treated with MpB and LpB. The occurrence of beauvericin, however, did not change. Furthermore, equisetin and sterigmatocystin appeared in potatoes treated with MpB (Table 1). Sterigmatocystin is, however, a cytotoxic compound of *Aspergillus* origin [57], present in our research as a probable soil contamination.

To the best of our knowledge, this is the first study to determine the secondary metabolite profile and mycotoxin contamination of potatoes inoculated with *Alternaria* spp. The results for the metabolite profile of *Alternaria tenuissima* growing on MEA plates and potatoes are presented in Table 2. Nine metabolites were detected in pure *A. tenuissima* cultured on MEA plates. Among them, three are classified as *Alternaria* species mycotoxins: AOH, AME, and ALX-I [46,58,59], which expressed genotoxic and strongly cytotoxic effects for humans in micromolar concentrations [60]. *Alternaria* spp. mycotoxins, AOH and AME, are the most dangerous in terms of risk to human health [61]. ALX-II, related to ALX-I, was demonstrated as a highly effective and selective compound against the Ewing sarcoma cell lines [59]. The metabolite profile of potatoes infected with *A. tenuissima* also included nine compounds, two of which (AME and TX) are known as metabolites of *Alternaria* origin [46,58]. 

As a probable result of cross-contamination originated from the soil, the presence of three *Fusarium*-derived mycotoxins was detected. The treatment of the LpB biopreparation resulted in reducing the contamination of *Alternaria* mycotoxins, and samples were free from AME in comparison to control potatoes (Table 2). On the contrary, the presence of ALTCH, a mycotoxin related to ALX-I, was only observed in potatoes treated with GB and LpB and then inoculated with *A. tenuissima*. Mycotoxin secretion is induced by various stimuli that are still under investigation. It is already known that several secondary metabolites are formed as a fungal adaptation to stress, e.g., too low/high temperature and pH changes, UV light, limited availability of nutrients, or the presence of fungicides [62], most likely including the biological treatments used in our work. 

The metabolite profile of *Rhizoctonia solani* growing on MEA plates and potatoes is presented in Table 3. The production of mycotoxins by *R. solani* is still unknown; however, Li et al. [63] recently identified 3-methoxyphenylacetic acid as a phytotoxin that is linked to tobacco leaf necrosis. In our work, only two unspecific metabolites were determined in the pure culture of *R. solani* growing on the MEA plate. The metabolite profile of fungal culture growing on potatoes included 10 compounds, half of which are unspecific or of plant origin. Four compounds, bikaverin, beauvericin, fusaric acid, and sambutoxin, are a probable cause of cross-contamination with *Fusarium* moulds, originated from the environment in which potatoes were grown. *Fusarium* mycotoxins identified in control potato samples were also present in samples treated with biopreparations (Table 3).

The results for secondary metabolites of *Phoma exigua* growing on MEA plates and potatoes are presented in Table 4. Five metabolites were identified in the pure culture of *P. exigua* growing on MEA plates. Among them, only cytochalasin B was recognised as a mycotoxin of *Phoma* origin [64], while the other compounds were unspecific. The metabolite profile of potatoes inoculated with *P. exigua* was more complex and contained 14 compounds, including bikaverin and toxic cytochalasin B, but mostly other unspecific or compounds of plant, lichen, and bacterial origin. The MpB biopreparation had a positive influence on reducing potato contamination with *Phoma* mycotoxin cytochalasin B (Table 4). Samples treated with biopreparations, however, contained *Fusarium* spp. mycotoxins.

The metabolite profile of *Colletotrichum coccodes* growing on MEA plates and potatoes is presented in Table 5. The production of mycotoxins by *C. coccodes* is still unknown. Nine compounds were present in the pure culture of *C. coccodes* growing on MEA plates. They were mainly unspecific components with the exception of bacterial monocerin and cytochalasin E, originally isolated from the *Aspergillus* species [65], as a probable effect of cross-contamination. Eleven metabolites were identified in potatoes inoculated with *C. coccodes*, mostly unrelated to fungal activity, except for several compounds originally isolated from the *Fusarium* species, such as beauvericin, bikaverin, and deoxygerfelin (Table 5). Potato samples treated with biopreparations contained *Fusarium* spp. mycotoxins (fusaric acid, beauvericin, and sambutoxin). The presence of mycotoxins and other secondary metabolites that are not produced by the fungi inoculated in the potato originated from the environment, i.e., the soil where potatoes were grown.

Chaconine and solanine, common potato peel metabolites, were determined in all potato samples (Table 1, Table 2, Table 3, Table 4 and Table 5). Their concentration varied significantly across individual tubers. The results of LC–MS/MS analysis of the potato control sample indicated high mycotoxin contamination, especially characteristic of the *Fusarium* spp. (Table 1, Table 2, Table 3, Table 4 and Table 5; Table S1), even if visible symptoms of infection on the tubers were not observed.

### 3.2. Growth and Physiological Parameter Analysis

#### 3.2.1. Germination of Seed Potatoes and Stem and Root Development

Untreated seed potatoes (control; C), stored at 4 °C from harvest until 1 April 2022 and then for 2 weeks at 15 °C and 80% RH, were characterised by high healthiness, typical phenotypic characteristics, and high turgor. After planting in the ground at 20 °C on 14 April, they germinated at 100%. Seed potatoes treated with biological agents, such as aqueous garlic extract biopreparation (GB), *Metschnikowia pulcherrima* TK1 biopreparation (MpB), and *Lactiplantibacillus plantarum* KB2 LAB 03 biopreparation (LpB), and infected with a mixture of phytopathogens, did not lose the turgor, germination capacity, and phenotypic properties of the mother tubers. During planting into the substrate, the seed potatoes did not show any mechanical damage, and no visible symptoms of disease were observed. This indicates that the treatment of seed potatoes with the biological preparations used and the inoculation of phytopathogens resulted in their proper germination, without damaging the sprouts mechanically or pathogenically, as was shown by Kirk and Gachango [66] for *Fusarium* contamination.

**Figure 1:**
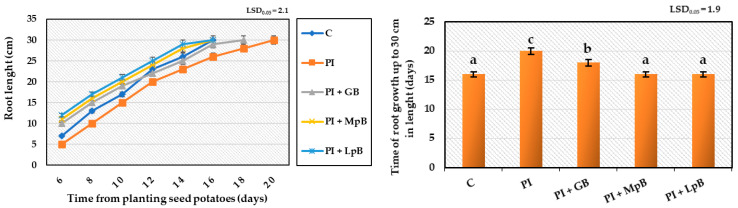
Growth kinetics and growth period of roots up to 30 cm in length, obtained from Impresja seed potatoes treated with biopreparations and then inoculated with phytopathogens. C—control potato, not treated with any biopreparations and not inoculated with pathogens; PI—potato sample only inoculated with pathogens; PI + GB—potato sample treated with aqueous garlic extract biopreparation and inoculated with pathogens; PI + MpB—potato sample treated with *M. pulcherrima* TK1 biopreparation and inoculated with pathogens; PI + LpB—potato sample treated with *L. plantarum* KB2 LAB 03 biopreparation and inoculated with pathogens. Means marked with the same letters do not differ statistically at the significance level *p* = 0.05; LSD—least significant difference.

**Figure 2:**
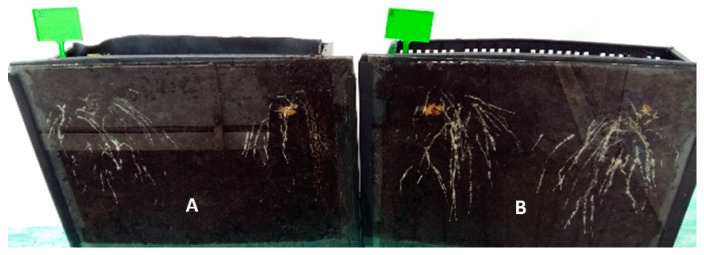
Soil profile overgrown by developing root system obtained from seed potatoes: (**A**) infected with phytopathogens; (**B**) biologically treated and then infected with phytopathogens.

**Figure 3:**
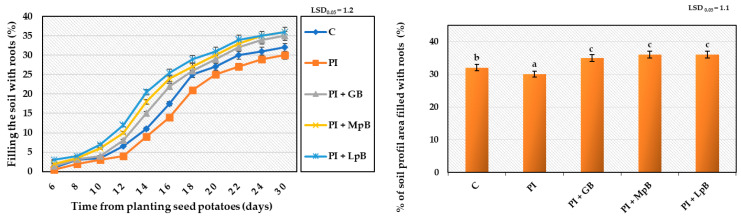
Kinetics of filling the soil profile with roots and final filling after 30 days from planting Impresja seed potatoes treated with biopreparations and inoculated with phytopathogens. C—control potato, not treated with any biopreparations and not inoculated with pathogens; PI—potato sample only inoculated with pathogens; PI + GB—potato sample treated with aqueous garlic extract biopreparation and inoculated with pathogens; PI + MpB—potato sample treated with *M. pulcherrima* TK1 biopreparation and inoculated with pathogens; PI + LpB—potato sample treated with *L. plantarum* KB2 LAB 03 biopreparation and inoculated with pathogens. Means marked with the same letters (a–c) do not differ statistically at the significance level *p* = 0.05; LSD—least significant difference.

The research showed that the treatment of seed potatoes with biological agents prevented the development of diseases initiated by the inoculated mixtures of phytopathogens. The pathogenic activity of phytopathogens and the fungistatic activity of the biopreparations applied to seed potatoes affected the growth, development, and physiological activity of stems and roots obtained from them to a different extent. The biggest impact of these treatments was observed in the growth and development of the root system, assessed by the dynamics of elongation of individual roots, filling the soil profile with them (Figure 1, Figure 2 and Figure 3). Inoculation of seed potatoes with a mixture of phytopathogens (PI) significantly delayed and reduced the growth dynamics of particular roots and their filling of the soil profile in comparison to the control sample without phytopathogens (C) and biological agent treatment. The root growth up to 30 cm in length lasted 20 days for the PI sample in comparison to 16 days in the case of the C sample. The percentage of soil profile area filled with roots was 30% for the PI sample and 32% for the C sample. Seed potato treatment with biopreparations before applying the mixture of phytopathogens had a significantly positive effect on the growth and development of the root system (Figure 1, Figure 2 and Figure 3). In particular, this beneficial effect was achieved for the treatment of seed potatoes with LpB and MpB. Root growth up to 30 cm in length lasted for 16 days for the MpB and LpB samples, and this was the same for control sample C. The percentage of soil profile area filled with roots was 36% for both the MpB and the LpB samples. The use of MpB and LpB biopreparations allowed entirely eliminating the negative effect of phytopathogen development on root growth and even stimulating it when the soil profile filling with roots was taken into account. In addition, Hamed et al. [67] and Abdel-Aziz et al. [22] observed a positive effect of lactic acid bacteria treatment on root development in tomato plants (increases of 216–358% and 271–305% over the control, respectively). 

The treatment of seed potatoes with GB resulted in the prevention of the negative impact of the applied phytopathogens, but to a lesser extent. These results indicate the high sensitivity of the root system to various stimuli, including the treatment of seed potatoes with biological compounds and pathogenic mycoflora, which significantly affected the growth, development, and yield of plants (Figure 1, Figure 2 and Figure 3). The biopreparations used for seed potato biological protection and the content of inoculated mixtures of phytopathogens caused different growth dynamics of the root system, indicating the possibility of a degree of improvement or deterioration of the physiological condition of sprouts. Compared to stems, resting and sprouting sprouts in moist soil are particularly exposed to the phytopathogenic activity of pathogens that completely or partially contaminate the surface of seed potatoes, which can then infect other parts of the plant. For this reason, as well as earlier development compared to other plant organs, root growth may be a useful marker for the assessment of the pathogenic activity of mycoflora contaminating seed potatoes [66,68]. The high sensitivity of the root system to the various factors applied to seeds and plants, and the usefulness of this test in assessing the reaction of plants to these treatments have also been indicated in earlier studies by the authors. This method of testing plant reactions to stimuli is noninvasive and more accurate, in addition to allowing a quicker indication of plant response to treatments than, for example, measurements of the height and physiological activity of stems, whose growth depends on the development of the roots [40,69,70,71].

**Figure 4:**
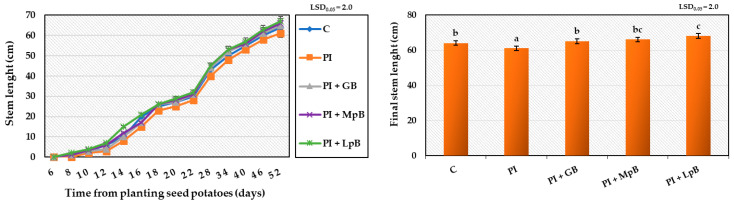
The kinetics of stem growth and their final length after 52 days from planting Impresja seed potatoes treated with biopreparations and inoculated with phytopathogens. C—control potato, not treated with any biopreparations and not inoculated with pathogens; PI—potato sample only inoculated with pathogens; PI + GB—potato sample treated with aqueous garlic extract biopreparation and inoculated with pathogens; PI + MpB—potato sample treated with *M. pulcherrima* TK1 biopreparation and inoculated with pathogens; PI + LpB—potato sample treated with *L. plantarum* KB2 LAB 03 biopreparation and inoculated with pathogens. Means marked with the same letters (a–c) do not differ statistically at the significance level *p* = 0.05; LSD—least significant difference.

**Figure 5:**
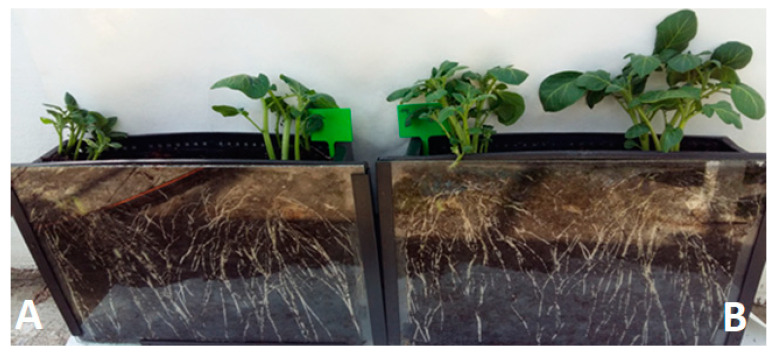
Plants obtained from seed potatoes: (**A**) infected with phytopathogens; (**B**) biologically treated and then infected with phytopathogens.

Treatment of seed potatoes with GB, MpB, and LpB, and then infecting them with a mixture of phytopathogens affected the growth and physiological activity of the stems (Figure 4 and Figure 5), but to a much lesser extent than the development of the root system. The plants obtained from untreated seed potatoes (C) began to emerge 6 days after planting and grew for the next 46 days (52 days in total), reaching a final height of 64 cm and forming flower buds. The stems obtained from seed potatoes infected with phytopathogens (PI) grew slightly more slowly than the control samples (final stem length was 61 cm), albeit without adversely affecting their flowering and vigour. Treatment of seed potatoes with biological agents in the period prior to inoculation with phytopathogens prevented the negative impact of microorganisms, which led to more dynamic stem growth. As observed for the roots, treatment of seed potatoes with MpB (final stem length was 66 cm) and LpB (final stem length was 68 cm) was the most beneficial. The obtained results indicate that the applied biological shield of biopreparations and the inoculation of phytopathogens into seed potatoes may affect the growth of stems. This effect, however, depends on the degree of infection of seed potato sprouts with pathogens and the degree of infection of the growing stems. In this case, the degree of sprout infection seems not to be sufficiently dangerous, leading to a slight decrease in stem growth dynamics, and it was clearly dependent on the biopreparations and the mixture of phytopathogens applied to the seed potatoes [66,68].

#### 3.2.2. Gas Exchange in Leaves and Index of Chlorophyll Content in Leaves

**Figure 6:**
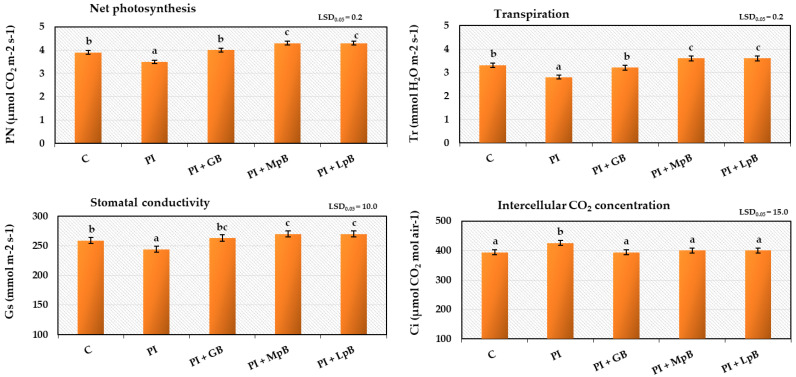
Gas exchange in leaves obtained from Impresja seed potatoes treated with biopreparations and inoculated with phytopathogens. C—control potato, not treated with any biopreparations and not inoculated with pathogens; PI—potato sample only inoculated with pathogens; PI + GB—potato sample treated with aqueous garlic extract biopreparation and inoculated with pathogens; PI + MpB—potato sample treated with *M. pulcherrima* TK1 biopreparation and inoculated with pathogens; PI + LpB—potato sample treated with *L. plantarum* KB2 LAB 03 biopreparation and inoculated with pathogens. Means marked with the same letters (a–c) do not differ statistically at the significance level *p* = 0.05; LSD—least significant difference.

**Figure 7:**
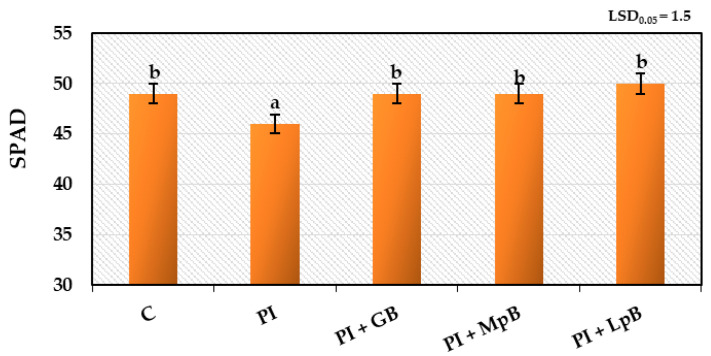
Index of chlorophyll content in leaves obtained from Impresja seed potatoes treated with biopreparations and inoculated with phytopathogens. C—control potato, not treated with any biopreparations and not inoculated with pathogens; PI—potato sample only inoculated with pathogens; PI + GB—potato sample treated with aqueous garlic extract biopreparation and inoculated with pathogens; PI + MpB—potato sample treated with *M. pulcherrima* TK1 biopreparation and inoculated with pathogens; PI + LpB—potato sample treated with *L. plantarum* KB2 LAB 03 biopreparation and inoculated with pathogens. Means marked with the same letters (a,b) do not differ statistically at the significance level *p* = 0.05; LSD—least significant difference.

The study of gas exchange activity in leaves (net photosynthesis, transpiration, stomatal conductivity, and intercellular CO_2_ content) and the index of chlorophyll contentconfirmed the correlation between the methods of biological treatment of seed potatoes and the development of roots and stems, as discussed in Section 3.2.1. (Figure 6 and Figure 7). Inoculation of seed potatoes with phytopathogens adversely affected gas exchange and the index of chlorophyll content in leaves. Treatment of seed potatoes with biopreparations, especially MpB and LpB, significantly increased this activity. The obtained results confirmed the close, verified by the literature, relationship between the tested physiological activity and plant growth, which depends on gas exchange in the leaves and the content of the photosynthetic pigment [72]. These parameters are important in the case of the potato, whose leaves did not show pathogenic symptoms visible to the naked eye, while their measurements demonstrated a beneficial effect of the appropriate biopreparations and a harmful pathogenic effect of phytopathogens [73].

The applied methods of biological treatment of the Impresja variant of seed potatoes had a similar effect on the quality of stems and leaves, assessed using a five-point rating scale. During the 52 days of the study, no significant changes in their vigour and colouration were found. 

## 4. Conclusions

Biological treatment exhibited an overall positive effect on the inhibition of metabolite production by the tested fungi on potatoes. In comparison to the control potatoes, after MpB treatment, aurofusarin, sambutoxin, and fusaric acid were not present in *Fusarium*-contaminated potatoes, while cytochalasin B was not formed in *Phoma*-inoculated samples. In potatoes treated with LpB and contaminated by *Alternaria* spores, no presence of alternariolmethylether was noted. Likewise, fusaric acid and sambutoxin were not determined in *Fusarium*-inoculated potatoes. In the GB treatment, aurofusarin was absent in *Fusarium*-contaminated potatoes in comparison to the control. Treatment with biopreparations, however, did not reduce the formation of all mycotoxins. Some mycotoxins were observed exclusively in samples treated with LpB (alteichin in the sample inoculated with *A. tenuissima*), MpB (equisetin and sterigmatocystin in the sample inoculated with *F. sambucinum*), and GB (alteichin in the sample inoculated with *A. tenuissima*). It is worth mentioning that biological treatment with MpB, LpB, and GB increased the growth of potato roots and stems, as well as improved the basic parameters of potato physiology: gas exchange and the index of chlorophyll content. Knowledge about the cellular biology underlying interactions between microbes in their natural environments is still relatively scarce. The tested bioprotective agents are environmentally friendly and suitable for use at an early stage for reducing contamination caused by fungi. Our promising results, however, require further examination in order to determine the optimal composition of biopreparations for the effective and long-lasting biological control of potato pathogens.

## Figures and Tables

**Table 1:** Secondary metabolites of *Fusarium sambucinum* cultured on MEA plates and potatoes treated with biopreparations.

Components	Concentration Mean ± SD [ng/g]				
	*F. sambucinum* Culture on MEA Plate (*n* = 3)	*F. sambucinum* Culture on Potato (*n* = 3)	Potatoes Treated with Biological Preparations and Infected with *F. sambucinum*		
			GB (*n* = 3)	LpB (*n* = 3)	MpB (*n* = 3)
Abscisic acid	<LOD ^b^	<LOD ^b^	<LOD ^b^	<LOD ^b^	30.2 ± 20.4 ^a^
Acuminatum B	20,500 ± 584 ^a^	<LOD ^b^	<LOD ^b^	<LOD ^b^	<LOD ^b^
Acuminatum C	9380 ± 862 ^a^	<LOD ^b^	<LOD ^b^	<LOD ^b^	<LOD ^b^
Asterric acid	<LOD ^a^	83.3 ± 112 ^a^	<LOD ^a^	50.9 ± 9.73 ^a^	<LOD ^a^
Aurofusarin	<LOD ^b^	7.27 ± 2.49 ^a^	<LOD ^b^	3.50 ± 0.0718 ^a^	<LOD ^b^
Beauvericin ^1^	6.3 ± 2 ^a^	27.5 ± 17.8 ^a^	73.8 ± 63.6 ^a^	11.8 ± 15.9 ^a^	2.21 ± 1.55 ^a^
Bikaverin ^1^	<LOD ^a^	505 ± 510 ^a^	5.04 ± 1.40 ^a^	<LOD ^a^	31.1 ± 29.5 ^a^
Brevianamide F ^2^	20.8 ± 1.13 ^a^	<LOD ^b^	<LOD ^b^	<LOD ^b^	<LOD ^b^
Chaconin	<LOD ^a^	<LOD ^a^	1320 ± 1070 ^a^	2240 ± 1740 ^ab^	5560 ± 2080 ^b^
Chrysogin	14.8 ± 4.59 ^a^	<LOD ^b^	<LOD ^b^	<LOD ^b^	<LOD ^b^
Citreorosein	<LOD ^b^	<LOD ^b^	<LOD ^b^	1.54 ± 0.128 ^a^	<LOD ^b^
Cordycepin ^1,2^	<LOD ^a^	65.6 ± 11 ^a^	106 ± 120 ^a^	169 ± 144 ^a^	43.5 ± 7.32 ^a^
cyclo(L-Pro-L-Tyr) ^2^	67.2 ± 14.9 ^a^	<LOD ^b^	<LOD ^b^	<LOD ^b^	<LOD ^b^
cyclo(L-Pro-L-Val) ^2^	216 ± 10.7 ^a^	<LOD ^b^	<LOD ^b^	<LOD ^b^	<LOD ^b^
Cyclosporin A	<LOD ^b^	<LOD ^b^	948 ± 407 ^a^	<LOD ^b^	<LOD ^b^
Cyclosporin B	<LOD ^b^	<LOD ^b^	114 ± 80.2 ^a^	<LOD ^b^	<LOD ^b^
Cyclosporin D	<LOD ^b^	<LOD ^b^	50.8 ± 27.4 ^a^	<LOD ^b^	<LOD ^b^
Cyclosporin H	<LOD ^b^	<LOD ^b^	783 ± 338 ^a^	<LOD ^b^	<LOD ^b^
Deoxygerfelin ^1^	<LOD ^a^	34.3 ± 33.2 ^a^	<LOD ^a^	0.668 ± 0.679 ^a^	10.8 ± 10.6 ^a^
Diacetoxyscirpenol	147.1 ± 17.3 ^a^	<LOD ^b^	<LOD ^b^	<LOD ^b^	<LOD ^b^
Dinactin	<LOD^b^	<LOD ^b^	<LOD ^b^	<LOD ^b^	3.04 ± 1.72 ^a^
Emodin ^1^	<LOD ^a^	4.31 ± 2.90 ^a^	<LOD ^a^	1.65 ± 2.35 ^a^	7.64 ± 7.36 ^a^
Endocrocin	<LOD ^ab^	203 ± 113 ^cd^	<LOD ^ae^	31.9 ± 3.61 ^ace^	63.7 ± 64.8 ^bde^
Equisetin	<LOD ^b^	<LOD ^b^	<LOD ^b^	<LOD ^b^	1.67 ± 1.07 ^a^
Fallacinol	<LOD ^b^	<LOD ^b^	<LOD ^b^	2.78 ± 2.08 ^a^	<LOD ^b^
Fusaric acid	<LOD ^a^	370 ± 133 ^a^	7870 ± 6990 ^a^	<LOD ^a^	<LOD ^a^
Gibepyron D	<LOD ^b^	<LOD ^b^	46.5 ± 34.3 ^a^	<LOD ^b^	<LOD ^b^
Iso-Rhodoptilometrin	<LOD ^a^	24.4 ± 21 ^a^	<LOD ^a^	3.92 ± 2.56 ^a^	4.87 ± 5.47 ^a^
Isosulochrin ^1^	<LOD ^a^	25.9 ± 26.8 ^a^	<LOD ^a^	28.2 ± 40.7 ^a^	<LOD ^a^
Lecanoric acid ^1^	<LOD ^a^	232 ± 262 ^a^	<LOD ^a^	27.6 ± 34.1 ^a^	218 ± 305 ^a^
Monactin	<LOD ^b^	<LOD ^b^	<LOD ^b^	<LOD ^b^	26.7 ± 256 ^a^
Nonactin	<LOD ^a^	<LOD ^a^	<LOD ^a^	<LOD ^a^	2.61 ± 2.04 ^a^
Radicinin	<LOD ^b^	<LOD ^b^	1.14 ± 0.16 ^a^	<LOD ^b^	<LOD ^b^
Sambutoxin	<LOD ^b^	0.29 ± 0.15 ^a^	0.0220 ± 0.0113 ^b^	<LOD ^b^	<LOD ^b^
Secalonic acid D	<LOD ^a^	<LOD ^a^	<LOD ^a^	3.53 ± 1.93 ^b^	2.57 ± 2.24 ^ab^
Solanin ^1^	<LOD ^a^	3250 ± 2000 ^ab^	462 ± 393 ^a^	1970 ± 1670 ^ab^	7820 ± 5010 ^b^
Sterigmatocystin	<LOD ^b^	<LOD ^b^	<LOD ^b^	<LOD ^b^	0.238 ± 0.228 ^a^
Sulochrin ^1^	<LOD ^a^	60.9 ± 68.2 ^a^	<LOD ^a^	91.9 ± 108 ^a^	<LOD ^a^
Terragine ^1^	<LOD ^a^	1310 ± 795 ^a^	2830 ± 3080 ^a^	8350 ± 13,300 ^a^	944 ± 181 ^a^
Tryptophol ^1,2^	<LOD ^b^	<LOD ^b^	<LOD ^b^	<LOD ^b^	14.2 ± 4.75 ^a^
Violaceol I	<LOD ^a^	1670 ± 1970 ^a^	<LOD ^a^	1120 ± 1380 ^a^	6080 ± 7270 ^a^
Violaceol II ^1^	<LOD ^a^	17.7 ± 20.3 ^a^	<LOD ^a^	<LOD ^a^	<LOD ^a^
Vulpinic acid	<LOD ^b^	<LOD ^b^	<LOD ^b^	<LOD ^b^	0.0993 ± 0.0542 ^a^
Xanthoquinoidin A1	<LOD ^a^	<LOD ^a^	<LOD ^a^	<LOD ^a^	24.8 ± 18.5 ^a^

^1^ Also present in potato control sample; the exact concentration is presented in Table S1. ^2^ Also present in MEA control sample; the exact concentration is presented in Table S1. <LOD—below level of detection; GB—aqueous garlic extract biopreparation; MpB—*M. pulcherrima* TK1 biopreparation; LpB—*L. plantarum* KB2 LAB 03 biopreparation. Underlined compounds are specific for a given mould. Decreases in the concentration of components in GB, LpB, and MpB samples compared to potatoes inoculated with mould and not treated with biopreparation are blue shading. *n*—number of replicates. Results with different letters (a–e) for each compound are significantly different (Tukey’s test, α = 0.05). * Statistically significant differences between the GB/LpB/MpB samples and the fungal culture on potato samples (one-way ANOVA, *p* < 0.05).

**Table 2:** Secondary metabolites of *Alternaria tenuissima* growing on MEA plates and potatoes treated with biopreparations.

Components	Concentration Mean ± SD [ng/g]				
	*A. tenuissima* Culture on MEA Plate (*n* = 3)	*A. tenuissima* Culture on Potato (*n* = 3)	Potatoes Treated with Biological Preparations and Infected with *A. tenuissima*		
			GB (*n* = 3)	LpB (*n* = 3)	MpB (*n* = 3)
4-Hydroxyalternariol	32.7 ± 10.2 ^a^	<LOD ^b^	<LOD ^b^	<LOD ^b^	<LOD ^b^
Abscisic acid	<LOD ^a^	<LOD ^a^	40.6 ± 10.2 ^d^	21.9 ± 14.4 ^ad^	33.9 ± 4.57 ^d^
Alteichin	<LOD ^c^	<LOD ^c^	8.98 ± 1.14 ^a^	3.97 ± 1.83 ^b^	<LOD ^c^
Alternariol (AOH)	935 ± 90.7 ^a^	<LOD ^b^	11.8 ± 12.2 ^b^	<LOD ^b^	0.451 ± 0.222 ^b^
Alternariolmethylether (AME)	18.6 ± 4.54 ^a^	0.57 ± 0.55 ^b^	11.4 ± 12.6 ^ab^	<LOD ^b^	0.889 ± 0.352 ^b^
Altertoxin-I (ALX-1)	1030 ± 9.32 ^a^	<LOD ^c^	15.4 ± 2.79 ^b^	2.98 ± 0.85 ^bc^	5.45 ± 3.44 ^bc^
Asterric acid	<LOD ^a^	<LOD ^a^	58.3 ± 75.1 ^a^	192.5 ± 148.6 ^a^	91.7 ± 111 ^a^
Beauvericin^1^	<LOD ^a^	46.3 ± 68.6 ^a^	8.98 ± 8.08 ^a^	6.41 ± 6.18 ^a^	4.91 ± 3.23 ^a^
Bikaverin^1^	<LOD ^b^	16.6 ± 19.2 ^b^	265 ± 79.4 ^a^	78.1 ± 109.2 ^b^	86.9 ± 53.8 ^b^
Brevianamid F ^2^	9.36 ± 0.353 ^a^	<LOD ^b^	<LOD ^b^	<LOD ^b^	<LOD ^b^
Chaconin	<LOD ^a^	<LOD ^a^	5710 ± 700 ^b^	5460 ± 2060 ^b^	7680 ± 2510 ^b^
Citreorosein	<LOD ^b^	<LOD ^b^	1.87 ± 1.54 ^ab^	2.34 ± 0.20 ^ab^	0.993 ± 0.0613 ^ab^
Cordycepin ^1,2^	13.8 ± 1.68 ^a^	128 ± 120 ^a^	44.0 ± 4.51 ^a^	50.6 ± 7.87 ^a^	32.9 ± 4.91 ^a^
cyclo(L-Leu-L-Pro)	4.57 ± 1.54 ^a^	<LOD ^b^	<LOD ^b^	<LOD ^b^	<LOD ^b^
cyclo(L-Pro-L-Tyr) ^2^	58.6 ± 10.2 ^a^	<LOD ^b^	<LOD ^b^	<LOD ^b^	<LOD ^b^
Cytochalasin B	5.81 ± 3.27 ^a^	<LOD ^b^	<LOD ^b^	<LOD ^b^	<LOD ^b^
Deoxygerfelin ^1^	<LOD ^a^	<LOD ^a^	7.46 ± 7.71 ^a^	4.28 ± 5.23 ^a^	3.60 ± 2.96 ^a^
Dinactin	<LOD ^a^	<LOD ^a^	<LOD ^a^	<LOD ^a^	0.871 ± 1.08 ^a^
Emodin ^1^	<LOD ^b^	<LOD ^b^	2.92 ± 2.40 ^ab^	3.92 ± 1.20 ^a^	2.34 ± 1.58 ^ab^
Endocrocin	<LOD ^a^	<LOD ^a^	<LOD ^a^	54.7 ± 52.3 ^a^	<LOD ^a^
Fallacinol	<LOD ^b^	<LOD ^b^	1.20 ± 1.63 ^ab^	2.57 ± 0.38 ^a^	1.50 ± 0.975 ^ab^
Fusaric acid	<LOD ^a^	3820 ± 3850 ^a^	23.4 ± 15.1 ^a^	82.2 ± 65.2 ^a^	<LOD ^a^
Iso-Rhodoptilometrin	<LOD ^b^	0.478 ± 0.0493 ^bc^	0.91 ± 0.65 ^bc^	2.66 ± 1.49 ^ac^	1.07 ± 1.10 ^bc^
Isosulochrin ^1^	<LOD ^a^	<LOD ^a^	7.26 ± 9.28 ^a^	31.2 ± 39.1 ^a^	0.80 ± 0.70 ^a^
Lecanoric acid^1^	<LOD ^a^	<LOD ^a^	66.6 ± 42.3 ^a^	32.4 ± 24.1 ^a^	43.8 ± 56.9 ^a^
Sambutoxin	<LOD ^a^	0.0484 ± 0.0235 ^a^	0.0511 ± 0.045 ^a^	<LOD ^a^	0.0290 ± 0.0222 ^a^
Secalonic acid D	<LOD ^a^	<LOD ^a^	12.7 ± 13.5 ^a^	<LOD ^a^	15.6 ± 11.7 ^a^
Solanin ^1^	<LOD ^a^	31,500 ± 38,500 ^a^	7090 ± 1310 ^a^	9110 ± 6350 ^a^	19,100 ± 17,000 ^a^
Sulochrin ^1^	<LOD ^a^	<LOD ^a^	13.0 ± 17.1 ^a^	65.0 ± 82.9 ^a^	12.8 ± 15.0 ^a^
Tentoxin (TX)	<LOD ^a^	27.2 ± 30 ^a^	12.3 ± 6.79 ^a^	11.3 ± 5.52 ^a^	31.4 ± 45.2 ^a^
Terragine ^1^	<LOD ^a^	<LOD ^a^	<LOD ^a^	716 ± 179 ^b^	685 ± 25.5 ^b^
Tryptophol ^1,2^	<LOD ^a^	<LOD ^a^	18.0 ± 5.83 ^b^	9.42 ± 4.42 ^ab^	17.0 ± 2.95 ^b^
Violaceol I	<LOD ^a^	<LOD ^a^	5730 ± 6340 ^a^	1850 ± 1570 ^a^	8280 ± 13,100 ^a^

^1^ Also present in potato control sample; the exact concentration is presented in Table S1. ^2^ Also present in MEA control sample; the exact concentration is presented in Table S1. <LOD—below level of detection; GB—aqueous garlic extract biopreparation; *MpB—M. pulcherrima* TK1 biopreparation; *LpB—L*. *plantarum* KB2 LAB 03 biopreparation. Underlined compounds are specific for a given mould. Decreases in the concentration of components in GB, LpB, and MpB samples compared to potatoes inoculated with mould and not treated with biopreparation are blue shading. *n*—number of replicates. Results with different letters (a–d) for each compound are significantly different (Tukey’s test, α = 0.05). * Statistically significant differences between the GB/LpB/MpB samples and the fungal culture on potato samples (one-way ANOVA, *p* < 0.05).

**Table 3:** Secondary metabolites of *Rhizoctonia solani* growing on MEA plates and potatoes treated with biopreparations.

Components	Concentration Mean ± SD [ng/g]				
	*R. solani* Culture on MEA Plate (*n* = 3)	*R. solani* Culture on Potato (*n* = 3)	Potatoes Treated with Biological Preparations and Infected with *R. solani*		
			GB (*n* = 3)	LpB (*n* = 3)	MpB (*n* = 3)
Abscisic acid	<LOD ^a^	<LOD ^a^	30.8 ± 11.5 ^a^	37.5 ± 43.0 ^a^	27.2 ± 6.88 ^a^
Beauvericin A	<LOD ^b^	<LOD ^b^	0.33 ± 0.20 ^b^	1.27 ± 0.39 ^a^	0.299 ± 0.102 ^b^
Beauvericin ^1^	<LOD ^a^	36.1 ± 31.8 ^a^	7.15 ± 3.43 ^a^	53.7 ± 50.7 ^a^	24.5 ± 24.6 ^a^
Bikaverin ^1^	<LOD ^a^	23.6 ± 26.3 ^a^	116 ± 131 ^a^	48.8 ± 59.9 ^a^	164 ± 77.1 ^a^
Brevianamide F ^2^	2.47 ± 1.69 ^a^	<LOD ^b^	<LOD ^b^	<LOD ^b^	<LOD ^b^
Chaconine	<LOD ^b^	<LOD ^b^	9320 ± 3060 ^a^	7890 ± 1910 ^a^	7760 ± 3100 ^a^
Citreorosein	<LOD ^b^	<LOD ^b^	<LOD ^b^	<LOD ^b^	2.17 ± 0.49 ^a^
Cordycepin ^1,2^	<LOD ^cd^	124 ± 34.1 ^a^	43.9 ± 6.3 ^b c^	60.2 ± 28.1 ^b^	38.8 ± 17.3 ^bd^
cyclo(L-Leu-L-Pro)	7.97 ± 0.92 ^a^	<LOD ^b^	<LOD ^b^	<LOD ^b^	<LOD ^b^
Deoxygerfelin ^1^	<LOD ^b^	<LOD ^b^	11.8 ± 6.83 ^a^	1.03 ± 0.76 ^b^	6.53 ± 3.54 ^ab^
Dinactin	<LOD ^b^	<LOD ^b^	<LOD ^b^	0.71 ± 0.13 ^a^	0.16 ± 0.13 ^b^
Emodin ^1^	<LOD ^a^	<LOD ^a^	1.74 ± 0.89 ^a^	0.80 ± 0.77 ^a^	2.08 ± 1.26 ^a^
Endocrocin	<LOD ^a^	49 ± 49.3 ^a^	29.2 ± 7.76 ^a^	<LOD ^a^	39.4 ± 14.1 ^a^
Enniatin B	<LOD ^b^	<LOD ^b^	0.111 ± 0.0169 ^a^	<LOD ^b^	<LOD ^b^
Fallacinol	<LOD ^b^	<LOD ^b^	<LOD ^b^	<LOD ^b^	2.45 ± 0.403 ^a^
Fusaric acid	<LOD ^a^	2730 ± 3720 ^a^	<LOD ^a^	265.9 ± 116.3 ^a^	114 ± 63.1 ^a^
Iso-Rhodoptilometrin	<LOD ^b^	2.32 ± 2.52 ^ab^	2.53 ± 1.20 ^ab^	1.32 ± 1.14 ^b^	5.55 ± 1.37 ^a^
Isosulochrin ^1^	<LOD ^a^	<LOD ^a^	1.96 ± 2.25 ^a^	<LOD ^a^	9.69 ± 14.7 ^a^
Lecanoric acid ^1^	<LOD ^a^	<LOD ^a^	21 ± 22.6 ^a^	6.15 ± 4.10 ^a^	37.8 ± 34.8 ^a^
Monactin	<LOD ^b^	<LOD ^b^	3.37 ± 4.26 ^ab^	11.6 ± 5.40 ^a^	2.25 ± 1.91 ^ab^
Nonactin ^1^	<LOD ^b^	<LOD ^b^	0.60 ± 0.34 ^b^	1.43 ± 0.47 ^a^	<LOD ^b^
Sambutoxin	<LOD ^a^	0.45 ± 0.44 ^a^	<LOD ^a^	0.0691 ± 0.0620 ^a^	0.149 ± 0.133 ^a^
Secalonic acid D	<LOD ^a^	<LOD ^a^	<LOD ^a^	25.7 ± 17.8 ^a^	46.9 ± 45.3 ^a^
Solanine ^1^	<LOD ^a^	17,400 ± 10,300 ^a^	27,700 ± 17,500 ^a^	11,000 ± 5030 ^a^	22,500 ± 23,700 ^a^
Sulochrin ^1^	<LOD ^a^	<LOD ^a^	<LOD ^a^	<LOD ^a^	26.0 ± 31.4 ^a^
Terragine ^1^	<LOD ^a^	2640 ± 1800 ^a^	1780 ± 1270 ^a^	1520 ± 1480 ^a^	1390 ± 1360 ^a^
Tryptophol ^1,2^	<LOD ^b^	13.8 ± 5.52 ^ab^	15.0 ± 4.31 ^ab^	25.1 ± 15.1 ^a^	10.4 ± 5.16 ^a b^
Violaceol I	<LOD ^a^	<LOD ^a^	1870 ± 1720 ^a^	246 ± 29.2 ^a^	2070 ± 2480 ^a^
Violaceol II ^1^	<LOD ^a^	<LOD ^a^	41.6 ± 30.5 ^a^	10.2 ± 0.739 ^a^	30.7 ± 28.9 ^a^
Xanthoquinoidin A1	<LOD ^a^	<LOD ^a^	<LOD ^a^	22.9 ± 31.1 ^a^	<LOD ^a^

^1^ Also present in potato control sample; the exact concentration is presented in Table S1. ^2^ Also present in MEA control sample; the exact concentration is presented in Table S1. <LOD—below level of detection; GB—aqueous garlic extract biopreparation; *MpB—M. pulcherrima* TK1 biopreparation; *LpB—L*. *plantarum* KB2 LAB 03 biopreparation. Underlined compounds are specific for a given mould. Decreases in the concentration of components in GB, LpB, and MpB samples compared to potatoes inoculated with mould and not treated with biopreparation are blue shading. *n*—number of replicates. Results with different letters (a–d) for each compound are significantly different (Tukey’s test, α = 0.05).

**Table 4:** Secondary metabolites of *Phoma exigua* growing on MEA plates and potatoes treated with biopreparations.

Components	Concentration Mean ± SD [ng/g]				
	*P. exigua* Culture on MEA Plate (*n* = 3)	*P. exigua* Culture on Potato (*n* = 3)	Potatoes Treated with Biological Preparations and Infected with *P. exigua*		
			GB (*n* = 3)	LpB (*n* = 3)	MpB (*n* = 3)
Abscisic acid	<LOD ^c^	<LOD ^c^	33.2 ± 7.68 ^ab^	46.3 ± 7.62 ^a^	29.0 ± 3.03 ^b^
Beauvericin A	<LOD ^a^	<LOD ^a^	<LOD ^a^	0.334 ± 0.314 ^a^	<LOD ^a^
Beauvericin ^1^	<LOD ^a^	21.7 ± 32.1 ^a^	11.1 ± 1.27 ^a^	7.60 ± 11.9 ^a^	1.49 ± 0.461 ^a^
Bikaverin ^1^	<LOD ^a^	103 ± 142 ^a^	137 ± 55.4 ^a^	17.0 ± 5.17 ^a^	42.45 ± 2.96 ^a^
Brevianamide F ^2^	11.0 ± 0.67 ^a^	<LOD ^b^	<LOD ^b^	<LOD ^b^	<LOD ^b^
Chaconine	<LOD ^c^	<LOD ^c^	10,400 ± 662 ^ab^	7700 ± 1010 ^a^	13,500 ± 3720 ^b^
Citreorosein	<LOD ^c^	<LOD ^c^	2.34 ± 0.944 ^b^	4.70 ± 1.18 ^a^	<LOD ^c^
Cordycepin ^1,2^	2.76 ± 0.562 ^a^	86.0 ± 90.7 ^a^	35.4 ± 3.97 ^a^	23.2 ± 8.53 ^a^	38.1 ± 9.51 ^a^
cyclo(L-Pro-L-Tyr) ^2^	347 ± 42.9 ^a^	<LOD ^b^	<LOD ^b^	<LOD ^b^	<LOD ^b^
cyclo(L-Pro-L-Val) ^2^	269 ± 11.7 ^a^	<LOD ^b^	<LOD ^b^	<LOD ^b^	<LOD ^b^
Cytochalasin B	75,000 ± 5890 ^a^	238 ± 305 ^b^	9.98 ± 4.06 ^b^	552 ± 857 ^b^	<LOD ^b^
Deoxygerfelin ^1^	<LOD ^a^	1.83 ± 0.304 ^a^	7.36 ± 7.81 ^a^	2.26 ± 2.60 ^a^	3.41 ± 3.87 ^a^
Dinactin	<LOD ^a^	<LOD ^a^	1.51 ± 1.19 ^a^	0.186 ± 0.104 ^a^	1.37 ± 0.613 ^a^
Emodin ^1^	<LOD ^b^	0.830 ± 0.349 ^ab^	1.67 ± 1.11 ^ab^	1.17 ± 0.49 ^a^	2.07 ± 1.08 ^a b^
Endocrocin	<LOD ^b^	<LOD ^b^	<LOD ^b^	<LOD ^b^	49.5 ± 7.45 ^a^
Enniatin B	<LOD ^b^	<LOD ^b^	<LOD ^b^	9.30 ± 0.0543 ^a^	<LOD ^b^
Fallacinol	<LOD ^a^	<LOD ^a^	<LOD ^a^	2.07 ± 2.76 ^a^	<LOD ^a^
Iso-Rhodoptilometrin	<LOD ^b^	1.98 ± 1.67 ^ab^	0.98 ± 0.69 ^b^	1.05 ± 0.792 ^ab^	6.92 ± 4.23 ^a^
Isosulochrin ^1^	<LOD ^a^	<LOD ^a^	2.98 ± 3.73 ^a^	<LOD ^a^	1.42 ± 0.60 ^a^
Lecanoric acid ^1^	<LOD ^a^	8.51 ± 3.38 ^a^	34.2 ± 47.1 ^a^	16.0 ± 14.3 ^a^	5.44 ± 4.13 ^a^
Monactin	<LOD ^b^	<LOD ^b^	19.8 ± 14.2 ^a^	12.4 ± 2.56 ^ab^	6.05 ± 0.88 ^ab^
Nonactin ^1^	<LOD ^a^	0.97 ± 1.27 ^a^	2.42 ± 1.55 ^a^	1.40 ± 1.30 ^a^	0.52 ± 0.05 ^a^
Secalonic acid D	<LOD ^d^	<LOD ^cd^	5.94 ± 2.96 ^b c d^	5.89 ± 3.93 ^b^	23.6 ± 6.92 ^a^
Siccanol	<LOD ^b^	<LOD ^b^	69.8 ± 17.7 ^b^	190 ± 77.1 ^a^	<LOD ^b^
Solanine ^1^	<LOD ^b^	14,800 ± 20,800 ^ab^	31,000 ± 13,900 ^ab^	8890 ± 2700 ^ab^	44,300 ± 9400 ^a^
Sulochrin ^1^	<LOD ^b^	<LOD ^b^	<LOD ^b^	<LOD ^b^	2.07 ± 0.68 ^a^
Terragine ^1^	<LOD ^a^	2400 ± 3020 ^a^	1130 ± 518 ^a^	718 ± 320 ^a^	<LOD ^a^
Tryptophol ^1,2^	<LOD ^a^	69.9 ± 85.1 ^a^	12.7 ± 2.76 ^a^	18.5 ± 4.31 ^a^	18.3 ± 4.44 ^a^
Violaceol I	<LOD ^a^	<LOD ^a^	3460 ± 5550 ^a^	453 ± 606 ^a^	326 ± 279 ^a^
Violaceol II ^1^	<LOD ^a^	<LOD ^a^	93.9 ± 113 ^a^	547 ± 10.5 ^a^	<LOD ^a^
Xanthoquinoidin A1	<LOD ^a^	45.0 ± 57.3 ^a^	<LOD ^a^	<LOD ^a^	<LOD ^a^

^1^ Also present in potato control sample; the exact concentration is presented in Table S1. ^2^ Also present in MEA control sample; the exact concentration is presented in Table S1. <LOD—below level of detection; GB—aqueous garlic extract biopreparation; *MpB—M. pulcherrima* TK1 biopreparation; *LpB—L*. *plantarum* KB2 LAB 03 biopreparation. Underlined compounds are specific for a given mould. Decreases in the concentration of components in GB, LpB, and MpB samples compared to potatoes inoculated with mould and not treated with biopreparation are blue shading. *n*—number of replicates. Results with different letters (a–d) for each compound are significantly different (Tukey’s test, α = 0.05).

**Table 5:** Secondary metabolites of *Colletotrichum coccodes* growing on MEA plates and potatoes treated with biopreparations.

Components	Concentration Mean ± SD [ng/g]				
	*C. coccodes* Culture on MEA Plate (*n* = 3)	*C. coccodes* Culture on Potato (*n* = 3)	Potatoes Treated with Biological Preparations and Infected with *C. coccodes*		
			GB (*n* = 3)	LpB (*n* = 3)	MpB (*n* = 3)
Abscisic acid	<LOD ^a^	<LOD ^a^	<LOD ^a^	<LOD ^a^	26.4 ± 17.1 ^a^
Asterric acid	<LOD ^a^	<LOD ^a^	<LOD ^a^	<LOD ^a^	98.7 ± 158 ^a^
Beauvericin ^1^	<LOD ^a^	1.15 ± 0.984 ^a^	11.5 ± 9.68 ^a^	13.0 ± 13.9 ^a^	3.85 ± 4.31 ^a^
Bikaverin ^1^	<LOD ^b^	23.7 ± 23.4 ^ab^	99.4 ± 49.8 ^a^	44.5 ± 40.1 ^ab^	14.6 ± 1.92 ^b^
Brevianamid F ^2^	31.2 ± 2.81 ^a^	<LOD ^b^	<LOD ^b^	<LOD ^b^	<LOD ^b^
Chaconin	<LOD ^b^	<LOD ^b^	5110 ± 1100 ^a^	3480 ± 618 ^a^	5450 ± 2230 ^a^
Citreorosein	<LOD ^b^	<LOD ^b^	<LOD ^b^	<LOD ^b^	2.10 ± 0.479 ^a^
Cordycepin ^1,2^	<LOD ^b^	58.1 ± 23.5 ^ab^	92.3 ± 61.3 ^a^	88.3 ± 4.15 ^a^	33.2 ± 5.96 ^ab^
cyclo(L-Pro-L-Tyr) ^2^	490 ± 229 ^a^	<LOD ^b^	<LOD ^b^	<LOD ^b^	<LOD ^b^
cyclo(L-Pro-L-Val) ^2^	297 ± 81.3 ^a^	<LOD ^b^	<LOD ^b^	<LOD ^b^	<LOD ^b^
Cytochalasin E ^1^	38,000 ± 12,800 ^a^	<LOD ^b^	<LOD ^b^	<LOD ^b^	<LOD ^b^
Deoxygerfelin ^1^	<LOD ^a^	1.55 ± 0.359 ^a^	12.0 ± 14.4 ^a^	3.88 ± 5.11 ^a^	3.06 ± 3.24 ^a^
Dinactin	<LOD ^a^	<LOD ^a^	<LOD ^a^	<LOD ^a^	1.78 ± 1.71 ^a^
Emodin ^1^	6.13 ± 4.42 ^a^	<LOD ^a^	8.66 ± 11.5 ^a^	0.47 ± 0.29 ^a^	5.45 ± 3.98 ^a^
Endocrocin	370 ± 18.1 ^a^	<LOD ^b^	<LOD ^b^	28.2 ± 15.3 ^b^	23.8 ± 19.5 ^b^
Fallacinol	<LOD ^b^	<LOD ^b^	<LOD ^b^	<LOD ^b^	1.33 ± 1.07 ^a^
Fusaric acid	<LOD ^a^	<LOD ^a^	<LOD ^a^	4090 ± 5540 ^a^	<LOD ^a^
Iso-Rhodoptilometrin	0.384 ± 0.181 ^a^	0.413 ± 0.561 ^a^	8.96 ± 11.2 ^a^	1.36 ± 1.32 ^a^	1.88 ± 0.49 ^a^
Isosulochrin ^1^	<LOD ^a^	<LOD ^a^	<LOD ^a^	6.33 ± 10.4 ^a^	10.5 ± 12.3 ^a^
Lecanoric acid ^1^	<LOD ^a^	<LOD ^a^	31.1 ± 49.0 ^a^	13.40 ± 16.6 ^a^	27.6 ± 31.1 ^a^
Monactin	<LOD ^a^	10.3 ± 11.3 ^a^	<LOD ^a^	<LOD ^a^	18.6 ± 14.8 ^a^
Monocerin ^1^	1760 ± 487 ^a^	4.24 ± 5.32 ^b^	24.0 ± 9.74 ^b^	41.6 ± 33.3 ^b^	4.15 ± 3.62 ^b^
Nonactin ^1^	<LOD ^a^	1.11 ± 1.42 ^a^	<LOD ^a^	<LOD ^a^	1.59 ± 1.12 ^a^
Rosellichalasin ^1^	458 ± 65.0 ^a^	<LOD ^b^	<LOD ^b^	11.0 ± 0.65 ^b^	<LOD ^b^
Sambutoxin	<LOD ^a^	<LOD ^a^	<LOD ^a^	0.0807 ± 0.113 ^a^	<LOD ^a^
Solanin ^1^	<LOD ^a^	6140 ± 1690 ^a^	5250 ± 1870 ^a^	2950 ± 908 ^a^	9590 ± 9020 ^a^
Sulochrin ^1^	<LOD ^a^	<LOD ^a^	<LOD ^a^	<LOD ^a^	11.1 ± 15.6 ^a^
Terragine ^1^	<LOD ^b^	780 ± 830 ^ab^	3110 ± 1740 ^a^	1430 ± 156 ^ab^	1060 ± 578 ^ab^
Trichodimerol	<LOD ^b^	<LOD ^b^	<LOD ^b^	486 ± 298 ^a^	22.5 ± 18.9 ^b^
Trichotetronine	<LOD ^b^	<LOD ^b^	<LOD ^b^	40.3 ± 9.79 ^a^	<LOD ^b^
Tryptophol ^1,2^	<LOD ^c^	6.07 ± 2.16 ^ab^	10.4 ± 2.88 ^a^	<LOD ^c^	5.52 ± 0.89 ^b^
Violaceol I	<LOD ^a^	<LOD ^a^	2330 ± 3680 ^a^	1090 ± 1650 ^a^	1820 ± 2090 ^a^
Violaceol II ^1^	<LOD ^a^	<LOD ^a^	<LOD ^a^	38.8 ± 43.2 ^a^	47.0 ± 45.6 ^a^

^1^ Also present in potato control sample; the exact concentration is presented in Table S1. ^2^ Also present in MEA control sample; the exact concentration is presented in Table S1. <LOD—below level of detection; GB—aqueous garlic extract biopreparation; *MpB—M. pulcherrima* TK1 biopreparation; *LpB—L*. *plantarum* KB2 LAB 03 biopreparation. Underlined compounds are specific for a given mould. Decreases in the concentration of components in GB, LpB, and MpB samples compared to potatoes inoculated with mould and not treated with biopreparation are blue shading. *n*—number of replicates. Results with different letters (a–c) for each compound are significantly different (Tukey’s test, α = 0.05).

## Supplementary Materials

The following supporting information can be downloaded at https://www.mdpi.com/article/10.3390/ijerph20065221/s1: Table S1. LC–MS/MS secondary metabolite analysis of control samples—MEA and potato; Table S2. Analytical parameters of LC–MS/MS.
