# Supplementary material for: Metabolite Formation by Fungal Pathogens of Potatoes (Solanum tuberosum L.) in the Presence of Bioprotective Agents
Source: Int J Environ Res Public Health. 2023 Mar 22;20(6):5221. doi: 10.3390/ijerph20065221 (PMC10049107; doi:10.3390/ijerph20065221)
Supplement: Supplementary file 1 [file ijerph-20-05221-s001.zip › ijerph-2237714-supplementary.pdf]

Table S1. LC-MS/MS secondary metabolite analysis of control samples – MEA and potato.

Components	Concentration Mean ± SD [ng/g]	
	Control sample – MEA	Control sample – potato
Beauvericin	< LOD	0.686±0.820
Bikaverin	< LOD	4.24±4.93
Brevianamide F	530±10.7	< LOD
Cordycepin	13.8±13.1	44.3±5.34
cyclo(L-Leu-L-Pro)	< LOD	< LOD
cyclo(L-Pro-L-Tyr)	5710±127	< LOD
cyclo(L-Pro-L-Val)	3770±141	< LOD
Cytochalasin E	< LOD	14.2±12.7
Daidzein	1220±195	< LOD
Daidzin	132±96.7	< LOD
Deoxygerfelin	< LOD	14.3±18.2
Fellutanine A	25.8±2.51	< LOD
Genistein	1070±164	< LOD
Genistin	186±223	< LOD
Glycitein	302±32.4	< LOD
Glycitin	98.6±58.9	< LOD
Isosulochrin	< LOD	0.351±0.040
Lecanoric acid	< LOD	6.25±5.60
Monocerin	< LOD	45.0±45.5
Nonactin	< LOD	0.150±0.025
Rosellichalasin	< LOD	13.7±17.8
Rugulusovin	88.1±4.04	< LOD
Solanine	< LOD	133000±68200
Sulochrin	< LOD	0.399±0.540
Terragine	< LOD	363±66.7
Tryptophol	<LOD	4.34±2.70
Violaceol II	< LOD	0.609±0.184

<LOD – below limit of detection

Table S2. Analytical parameters of LC-MS/MS.

Compound	LOQ [ng/g]	LOD [ng/g]
4-Hydroxyalternariol	9.58	2.87
Abscisic acid	0.50	0.15
Acuminatum B	24.35	7.31
Acuminatum C	61.68	18.50
Alteichin	1.00	0.30
Alternariol	0.32	0.10
Alternariolmethylether	0.51	0.15
Altersetin	3.61	1.08
Altertoxin-I	4.86	1.46

Asterric acid	0.50	0.15
Aurofusarin	2.0	0.6
Beauvericin A	0.09	0.03
Beauvericin	0.09	0.03
Bikaverin	1.54	0.46
Brevianamide F	3.34	1.00
Chaconine	30	10
Chrysogin	1.86	0.56
Citreorosein	2.47	0.74
Cordycepin	0.6	0.2
cyclo(L-Leu-L-Pro)	2.20	0.66
cyclo(L-Pro-L-Tyr)	28.30	8.49
cyclo(L-Pro-L-Val)	3.88	1.17
Cyclosporin A	20.90	6.27
Cyclosporin B	3.67	1.10
Cyclosporin D	7.99	2.40
Cyclosporin H	2.76	0.83
Cytochalasin B	5.44	1.63
Cytochalasin E	1.43	0.43
Daidzein	5.6	1.7
Daidzin	5.6	1.7
Deoxygerfelin	0.07	0.02
Diacetoxyscirpenol	0.5	0.15
Dinactin	0.39	0.13
Emodin	0.35	0.11
Endocrocin	51.23	15.37
Enniatin B	0.04	0.01
Equisetin	2.30	0.69
Fallacinol	0.35	0.11
Fellutanine A	3.31	0.99
Fusaric acid	30	10
Genistein	8.1	2.7
Genistin	20	6
Gibepyron D	14.39	4.32
Glycitein	4.1	2.1
Glycitin	4.7	1.4
Iso-Rhodoptilometrin	0.12	0.03
Isosulochrin	0.19	0.06
Lecanoric acid	0.36	0.12
Monactin	0.63	0.19
Monocerin	0.20	0.06
Nonactin	0.18	0.05
Radicinin	1.64	0.60
Rosellichalasin	7.7	2.3
Rugulusovin	2.25	0.67
Sambutoxin	0.064	0.021

Secalonic acid D	2.86	0.86
Siccanol	100	30
Solanine	11	3.3
Sterigmatocystin	0.25	0.08
Sulochrin	0.52	0.16
Tentoxin	0.36	0.11
Terragine	100	30
Trichodimerol	0.91	0.27
Trichotetronine	7.47	2.24
Tryptophol	12.85	3.86
Violaceol I	12	3.6
Violaceol II	0.21	0.07
Vulpinic acid	0.17	0.06
Xanthoquinoidin A1	3	1

LOD – Limit of detection; LOQ – Limit of quantification;
